# The Novel Cytokine Interleukin-41/Meteorin-like Is Reduced in Diffuse Systemic Sclerosis

**DOI:** 10.3390/cells13141205

**Published:** 2024-07-17

**Authors:** Paul Freedman, Bettina Schock, Steven O’Reilly

**Affiliations:** 1 Department of Biosciences, Durham University, South Road, Durham DH1 3LE, UK; 2 The Wellcome-Wolfson Institute for Experimental Medicine, Queens University Belfast, Belfast BT9 7BL, UK

**Keywords:** systemic sclerosis, fibrosis, IL-41, cytokine, fibrotic

## Abstract

Systemic sclerosis (SSc) is an autoimmune connective tissue disease with a triad of features that include vascular abnormalities, inflammation and skin and lung fibrosis. At the core of the disease is the activation of myofibroblasts from quiescent fibroblasts and this can be modified by various cytokines. IL-41 is a recently described cytokine that was initially characterised as an adipokine as it was highly expressed in adipocytes and adipose tissue. However, it has recently been identified as being widely expressed and has immunomodulatory functions. This study examined the circulating levels of IL-41 and its expression in skin biopsies. We demonstrated significantly reduced levels of IL-41 in diffuse SSc that was also mirrored in the skin of SSc patients. AMPK has been proposed as a downstream target of IL-41, so we also measure mammalian target of rapamycin in skin and found that this is elevated in SSc patients. We speculate that IL-41 maybe an antifibrotic cytokine and its reduction may facilitate the activation of fibroblasts.

## 1. Introduction

Systemic sclerosis (SSc) is an autoimmune rheumatic connective tissue disease with a multifaceted burden including inflammation, vascular damage and subsequent skin fibrosis [1]. Disturbed immune regulation appears key, with alterations in multiple immune cells and autoantibodies against certain specific antigens. There is dysregulation of both innate and adaptive immunity [2,3] and multiple cytokines including IL-6 have been shown to be important in disease pathogenesis [4,5] and other cytokines such as those belonging to the Th2 family have also been shown to be important in disease [6]. Among all the autoimmune rheumatic diseases, SSc has the highest all-cause mortality, usually due to interstitial lung disease, and currently no disease-modifying treatment exists that targets skin fibrosis [7].

A novel recently described cytokine designated Interleukin-41 has also been called meteorin-like and is expressed in various cell types including monocytes [8], adipocytes and barrier tissues including skin and mucus membranes [9]. One of its primary functions is to stimulate whole-body cell expenditure [10], but it also has immunoregulatory functions [11]. In mouse macrophages, IL-41 is induced by the TLR4 agonist lipopolysaccharide (LPS) [11] and it regulates the release of multiple cytokines [11]. In the skin, it has high expression in fibroblasts and is upregulated in psoriasis and in the synovia of rheumatoid arthritis [8]. Multiple studies point to this cytokine having an immunomodulatory role in multiple inflammatory diseases including psoriatic arthritis [12], and most recently, IL-41 gene-ablated mice have been reported to have a reduced heart fibrosis in an experimental animal model of cardiac disease [13]. No data exist on IL-41 in systemic sclerosis; thus, the aim of this study was to determine IL-41 in diffuse SSc.

## 2. Materials and Methods

Eighteen patients with early diffuse SSc were involved in the study; this is a retrospective study of a single-centre study. Patients were defined as having early diffuse SSc where there were <2 years since the first non-Raynaud’s symptom. All patients fulfilled the American College of Rheumatology (ACR) criteria for a diagnosis of diffuse systemic sclerosis and full informed consent was provided from the patients involved. The study has full ethical approval with the local research ethics committee (REC) with approval no REC/13/NE/0089 and followed the Declaration of Helsinki guidelines. Healthy controls were age- and gender-matched and recruited from university students and staff; *n* = 18. There was 15 mL blood drawn from each donor’s arm and serum was isolated by centrifugation at 2000× *g* for 15 min. Serum was frozen immediately at −150 °C until thawed for downstream analysis.

A commercially available ELISA specific for IL-41 was used (D4050 R&D systems, Oxford, UK). Serum was thawed and diluted into the buffer and we followed the manufacturer’s instructions. All samples were run in triplicate and the plate was read using a Tecan Sunrise plate reader with a wavelength at 450 nm. The limit of detection (LOD) was 10 pg/mL and the limit of quantification (LOQ) was 25 pg/mL, with an intra-assay CV of 4%.

Skin biopsies were taken from the forearm using a 4 mm punch biopsy from affected skin on the anterior of the forearm in SSc patients, or in heathy controls’ forearms from healthy donor volunteers (*n* = 5). RNA was isolated from healthy and SSc skin biopsies and, after mechanical disruption using TRIzol and 1 μg converted to cDNA using Nanoscript 2 reverse transcriptase (Primer Design Ltd., Southampton, UK), Q-RT-PCR was performed using specific primers and SYBR^TM^ green (Sigma, Gillingham, UK). All data were normalised to the housekeeping gene 18S and relative differences were computed using the delta Ct method. No template control was ran as a control. Data are shown as fold change compared to healthy controls. Primers used: IL-41 F: 5′ GAGCTGGTTAGGAGGCACAG; Rev: 5′ AGGCTCGTGGGTAACTTGC; 18S F: 5′ CGAATG GCTCATTAA ATC AGT TAT GG 3′; Rev: 5′ TATTAGCTCTAGAATTACCACAGTTATCC3′; mTOR F: 5′ ACTGCTTTGAGGTCGCTATGA 3′; Rev: 5′ TTGCCTTTGGTATTTGTGTCC 3′. 

Statistical analysis was performed on the data. For sera IL-41 levels, the Mann–Whitney U test was performed and compared to HCs. For the gene expression analysis in the skin biopsies, Student’s *t* test was performed. For the correlation analysis between the serum IL-41 and mRSS skin score and IL-41 and mTOR gene expression, a two-tailed Pearson correlation analysis was performed with a *p* value ≤ 0.05 considered significant. All analysis was performed using GraphPad Prism^TM^ software version 10.

## 3. Results

**Figure 1:**
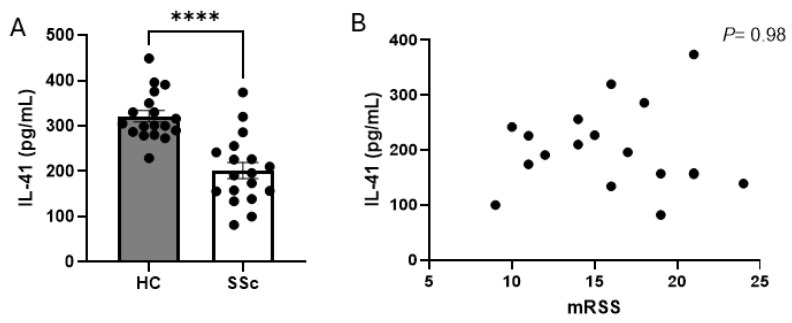
IL-41 is elevated in SSc serum. (**A**) Mean IL-41 levels in HC and diffuse SSc levels quantified by ELISA. Data are the mean and SEM from 18 donors; **** *p* = <0.0001 for Mann–Whitney U test. (**B**) No correlation between IL-41 and mRSS in SSc patients; r = −0.007, *p* = 0.98; Pearson correlation is two-tailed.

We sought to quantify the levels of IL-41 in the sera of SSc patients compared to healthy controls. There were 18 early diffuse SSc patients and 18 healthy controls included in the analysis. Early diffuse patients, defined as being 2 years or less from their first non-Raynaud’s symptom, were chosen as these were more “inflammatory”. Table 1 gives an overview of the patient demographics. The average age of SSc patients was 49.3 years old, whilst HC donors had an average age of 48 years old; there were 16 female and 2 male patients, which was the same gender split as in the HC group. It was found that, compared to healthy controls, SSc patients had significantly reduced levels of IL-41, with a mean for the HC donors of 321.2 (12.6 SEM) vs. 201.6 (17.8 SEM) pg/mL for SSc patients, with *p* ≤ 0.0001 for the Mann–Whitney U test; *n* = 18, Figure 1A. Next, we determined if there was a correlation between serum IL-41 concentration and the mRSS skin score. There was no correlation between these two variables; r^2^ = 0.00005, *p* = 0.98; *n* = 18, Figure 1B.

**Figure 2:**
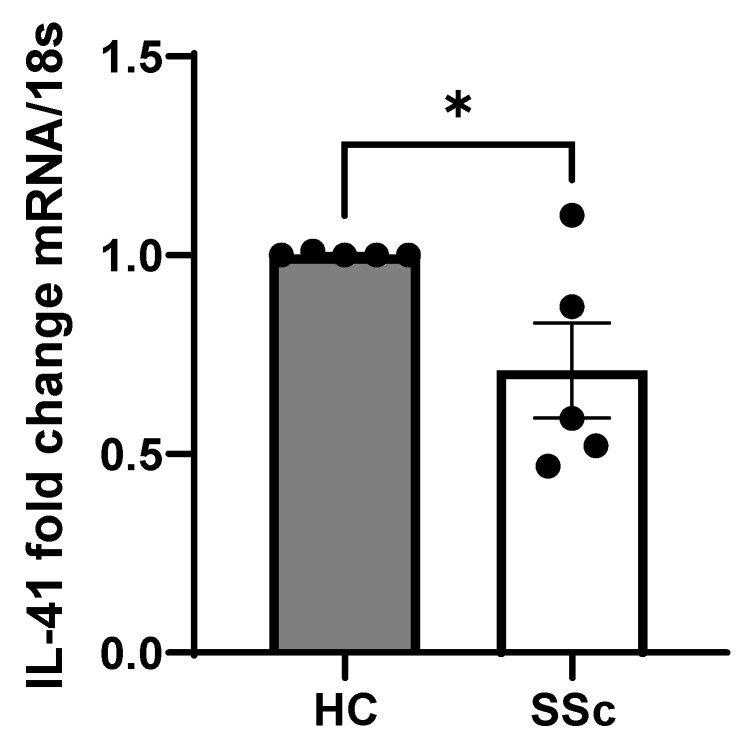
IL-41 is reduced in SSc skin. IL-41 was quantified in whole skin by qPCR and normalised to 18S gene and shown as fold change compared to HC. Data are the mean and SEM; * *p* = 0.04 for Students *t* test; *n* = 5.

**Figure 3:**
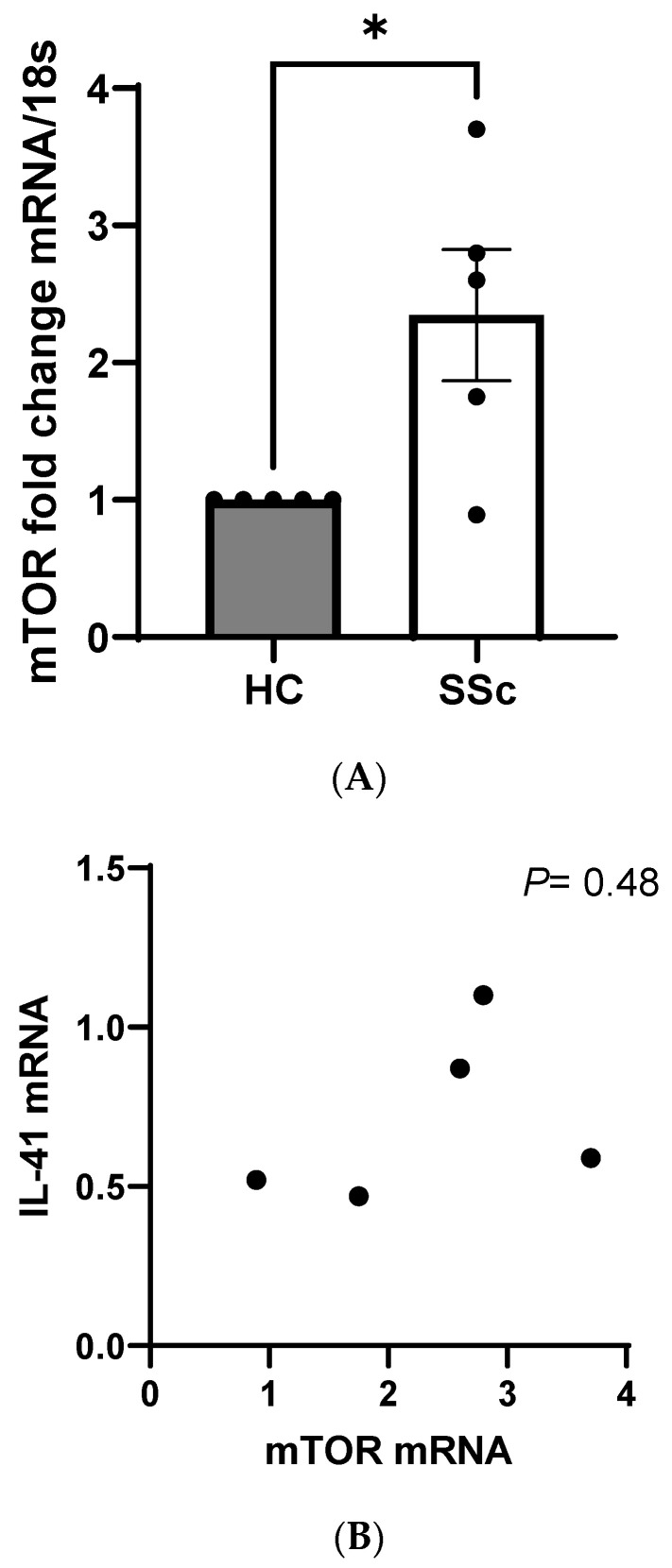
Elevated mTOR in SSc skin. (**A**) mRNA of mTOR in SSc or HC patient measured by qPCR and normalised to 18S gene and shown as fold change compared to HC. Data are the mean and SEM; * *p* = 0.022 for Student’s *t* test; *n* = 5. (**B**) Correlation analysis between IL-41 mRNA and mTOR mRNA expression; *p* = 0.48, r = 0.42; Pearsons correlation is two-tailed; *n* = 5.

Using whole skin biopsies isolated from five SSc donors we measured the mRNA levels of IL-41 in these samples. In the SSc samples, IL-41 was significantly reduced compared to the healthy controls, with a 1 vs. 0.71 mean fold change, *p* = 0.04 Student’s *t* test; *n* = 5 (Figure 2). Although no direct receptor has been identified for IL-41, it has been demonstrated that it is an activator of the critical nutrient kinase AMP-Kinase (AMPK) [14]. AMPK is a negative regulator of mammalian target of rapamycin (mTOR) [15]; thus, when AMPK activity is low, mTOR is high, and when AMPK activity is high, mTOR is low. We therefore measured in the same samples the levels of mTOR; this revealed that mTOR is significantly increased in the SSc skin samples compared to in healthy controls; *p* = 0.022 for Student’s *t* test, *n* = 5 (Figure 3A). We also examined the correlation between IL-41 gene-expressed mRNA and mTOR mRNA (Figure 3B). No correlation was observed (Figure 3B), with the following values for the Pearson correlation: *p* = 0.48, r = 0.42, r^2^ = 0.172 and *n* = 5.

## 4. Discussion

Although different studies report different results, it is mainly accepted that IL-41 is anti-inflammatory. In isolated in vitro endothelial cells, IL-41 was found to reduce reactive oxygen species (ROS) levels and reduce activation of the inflammasome component NLRP3, induced by palmitic acid stimulation [16]. Furthermore, Jung et al. demonstrated in both human endothelial cells and THP-1 monocytes that IL-41 attenuated tumour necrosis factor-α and MCP-1 release after LPS stimulation and that this was dependant on PPARγ and AMPK [14], demonstrating its key anti-inflammatory properties. Interestingly, the activation of PPARγ in systemic sclerosis fibroblasts shows strong antifibrotic activity in vitro and in vivo [17,18]. Indeed, the activation of AMPK alleviates pulmonary fibrosis [19,20], keloids [21], radiation-induced skin fibrosis [22] and animal models of SSc [23,24]. Furthermore, mTOR, which is usually repressed by AMPK upstream, was significantly elevated in our SSc skin samples, suggesting reduced AMPK activation possibly by reduced IL-41. However, we did not measure the phosphorylation status of AMPK in our skin tissue. The phosphorylation of AMPK is a better measurement of mTOR activation than overall amount; thus, it is difficult to draw definite conclusions. Further studies delineating the role of IL-41 and AMPK are needed. Indeed, in keloid fibroblasts, the inhibition of mTOR signalling with palomid592 reduced extracellular matrix deposition in vitro and in ex vivo keloid models [25]. In SSc dermal fibroblasts, the inhibition of mTOR was demonstrated to reduce collagen expression in vitro [26]. We found no correlation between IL-41 and mTOR mRNA levels. Furthermore, in two animal models of skin fibrosis, with the bleomycin model and the tight-skin mice, the classical inhibitor of mTOR rapamycin significantly suppressed fibrosis and was associated with reduced collagen deposition and pro-fibrotic cytokines [27]. We found significantly reduced serum IL-41 in SSc patients and in the skin. Reduced circulating IL-41 has been demonstrated in coronary heart disease [28] and, importantly, mice with genetic loss of IL-41 have exacerbated cardiac fibrosis [13]. The adenoviral overexpression of IL-41 in vivo protected the heart from fibrosis [13], and in vitro studies using a neutralising antibody for IL-41 reduced collagen expression in these mesenchymal cells. Indeed, it was recently demonstrated that the neutralisation via a specific antibody of IL-41 promoted allergic asthma in vivo with an increase in Th2-dominated cells and cytokines [29]. SSc is a Th2-dominated disease, with increased IL-4 and IL-13 [30,31]. This all suggests that IL-41 is an anti-fibrotic molecule and that its reduction would facilitate fibrosis in the correct environment. Its role in SSc is not clear and this is the first report of its expression in SSc and one can only speculate on its role, if any, in disease pathogenesis. It could be, given that IL-41 KO mice have ex-acerbated cardiac fibrosis, that this is an anti-fibrotic molecule. To date, this has not been tested in the classic model of systemic sclerosis, the bleomycin mouse model, but it could be predicted that in such a model skin fibrosis would be elevated compared to in wild-type mice exposed to bleomycin. Further studies to understand the mechanism of action of IL-41 and the possible role of AMPK and unidentified pathways are needed to understand its role, if any, in SSc and tissue fibrosis. 

## 5. Conclusions

In conclusion, we demonstrate reduced IL-41 in diffuse SSc and suggest that this may be an antifibrotic cytokine worthy of further investigation. The limitations of this study are the small sample size and its cross-sectional nature. Larger cohort studies are required.

## Figures and Tables

**Table 1:** Patient demographic data.

Patient Number	Age (Years)	Sex	Autoantibodies	mRSS	Treatment	ILD	DLCO%
**Patient_1**	48	F	Scl-70	9	None	N	85
**Patient_2**	54	F	Scl-70	16	None	N	82
**Patient_3**	51	F	RNA-polIII	10	None	N	89
**Patient_4**	66	F	Scl-70	16	None	Y	60
**Patient_5**	39	F	Scl-70	11	None	N	73
**Patient_6**	52	F	Scl-70	12	None	N	79
**Patient_7**	41	M	Scl-70	19	None	N	87
**Patient_8**	49	F	Scl-70	14	None	Y	57
**Patient_9**	55	F	RNA-polIII	17	None	Y	52
**Patient_10**	42	F	Scl-70	21	None	N	91
**Patient_11**	57	M	Scl-70	14	None	N	75
**Patient_12**	35	F	Scl-70	19	None	N	82
**Patient_13**	47	F	Scl-70	11	None	N	76
**Patient_14**	61	F	Scl-70	15	None	N	74
**Patient_15**	55	F	Scl-70	21	None	Y	50
**Patient_16**	37	F	Scl-70	18	None	N	78
**Patient_17**	47	F	Scl-70	21	None	N	83
**Patient_18**	51	F	Scl-70	24	None	N	87

## Data Availability

Available on request from corresponding author.
